# Supplementary material for: Can Presepsin Be Valuable in Reducing Unnecessary Antibiotic Exposure after Birth?
Source: Antibiotics (Basel). 2023 Apr 2;12(4):695. doi: 10.3390/antibiotics12040695 (PMC10134974; doi:10.3390/antibiotics12040695)

Supplemental Materials

Table S1. Plasma presepsin concentrations (pg/ml) at the different time points in term-born infants. All presepsin concentrations are presented as median [interquartile range].

	Uninfected controls (n=99)	EOS cases (n=65)
**T = Umbilical cord blood**	612 [520-873]	602 [559-602]
**T = Sepsis workup**	700 [563-890]	809 [614-1016]
**T = 3h**	740 [611-1027]	765 [642-1036]
**T = 6h**	742 [562-814]	833 [490-1561]
**T = 12h**	743 [506-956]	842 [639-1076]
**T = 24h**	479 [399-718]	701 [458-841]

Table S2. Diagnostic accuracy measures, including the area under the curve (AUC) and the sensitivity, specificity, positive predictive value (PPV) and negative predictive value (NPV) of Youden's index, along with 95% confidence intervals (CIs), in term-born infants.

	AUC (95% CI)	Sensitivity (95% CI)	Specificity (95% CI)	NPV (95% CI)	PPV (95% CI)
**T = Umbilical cord blood**	0.50 (0.10-0.99)	100 (16-100)	7 (10-93)	100 (100-100)	50 (28-73)
**T = Sepsis workup**	0.60 (0.50-0.70)	46 (32-61)	74 (63-83)	53 (38-69)	68 (57-78)

**T = 3h**	0.54 (0.36-0.72)	94 (72-100)	21 (7-42)	83 (39-98)	47(41-53)
**T = 6h**	0.62 (0.43-0.82)	59 (33-82)	71 (48-89)	68 (53-80)	63 (43-79)
**T = 12h**	0.60 (0.41-0.80)	92 (64-100)	32 (14-55)	88 (49-98)	44 (37-53)
**T = 24h**	0.65 (0.49-0.80)	65 (44-83)	26 (10-48)	40 (22-61)	50 (41-59)

Table S3. Plasma presepsin concentrations (pg/ml) at the different time points in preterm-born infants. All presepsin concentrations are presented as median [interquartile range].

	**Uninfected controls (n=154)**	**EOS cases (n=15)**
**T = Umbilical cord blood**	541 [393-742]	1563 [1127-1957]
**T = Sepsis workup**	643 [521-926]	1181 [879-1755]
**T = 3h**	640 [488-835]	1447 [738-2051]
**T = 6h**	648 [507-860]	886 [672-1448]
**T = 12h**	648 [497-924]	990 [673-1242]
**T = 24h**	599 [461-833]	1218 [830-2472]

Table S4. Diagnostic accuracy measures, including the area under the curve (AUC) and the sensitivity, specificity, positive predictive value (PPV) and negative predictive value (NPV) of Youden's index, along with 95% confidence intervals (CIs), in preterm-born infants.

	**AUC (95% CI)**	**Sensitivity (95% CI)**	**Specificity (95% CI)**	**NPV (95% CI)**	**PPV (95% CI)**
**T = Umbilical cord blood**	0.95 (0.88-1.00)	100 (40-100)	88 (76-96)	100 (100-100)	40 (24-59)

**T = Sepsis workup**	0.84 (0.73-0.95)	87 (60-98)	68 (58-77)	97 (90-100)	28 (16-43)
**T = 3h**	0.83 (0.70-0.96)	60 (26-88)	95 (87-99)	95 (90-98)	60 (34-82)
**T = 6h**	0.77 (0.64-0.90)	91 (59-99)	53 (42-63)	98 (89-99)	18 (14-22)
**T = 12h**	0.79 (0.66-0.92)	86 (42-99)	69 (58-79)	98 (90-99)	19 (13-27)
**T = 24h**	0.82 0.67-0.97)	88 (47-99)	70 (57-80)	98 (88-99)	25 (18-34)

Table S5. Area under the curve (AUC) along with 95% confidence intervals (CIs) for preterm-born infants divided into subgroups.

	Gestational age < 32^{+0} (n = 81 controls; 7 cases)	Gestational age 32^{+0} to 36^{+6} (n = 73 controls; 8 cases)
	AUC (95% CI)	AUC (95% CI)
**T = Umbilical cord**	1.00 (1.00-1.00)	0.82 (0.54-1.00)
**T = Sepsis workup**	0.98 (0.94-1.00)	0.73 (0.56-0.89)
**T = 3h**	0.86 (0.72-1.00)	0.80 (0.56-1.00)
**T = 6h**	0.84 (0.69-0.99)	0.66 (0.43-0.89)
**T = 12h**	0.83 (0.68-0.98)	0.69 (0.41-0.96)
**T = 24h**	0.92 (0.83-1.00)	0.50 (0.17-0.83)

Table S6. Maternal risk factors for early-onset sepsis as mentioned in the Dutch guideline.

Maternal risk factors	Red flag
Maternal parenteral antibiotic treatment with a suspicion of maternal sepsis during labor within 24 hours before or after birth	Yes
Suspected or confirmed infection in another baby in the case of a multiple pregnancy	Yes
Invasive group B streptococcal infection in a previous baby	No
Maternal group B streptococcal colonisation, bacteriuria or infection in the current pregnancy	No
Prelabor rupture of membranes for more than 24 hours before the onset of labor in term born infants	No
Rupture of membranes for more than 18 hours before a preterm birth	No
Pre-term birth following spontaneous labor before 37 weeks' gestation	No
Intrapartum maternal fever >38°C (regardless of epidural analgesia), or chorioamnionitis (suspected or proven)	No

Table S7. Neonatal clinical symptoms and risk factors as mentioned in the Dutch guideline.

Neonatal clinical symptoms	Red flag
Respiratory distress with onset more than four hours postpartum	Yes
Neonatal epileptic seizures	Yes
Need for mechanical ventilation	Yes
Signs of shock	Yes
Altered behaviour or responsiveness	No
Feeding difficulties (for example, feed refusal)	No
Apnoea and/or bradycardia	No

Signs of respiratory distress (including grunting, recession, tachypnoea)	No
Hypoxia (for example, central cyanosis or reduced oxygen saturation level)	No
Signs of neonatal encephalopathy	No
Need for cardiopulmonary resuscitation	No
Persistent pulmonary hypertension of newborns	No
Temperature abnormality (lower than 36°C or higher than 38°C) unexplained by environmental factors	No
Local signs of infection (for example, on the skin or eyes)	No

Figure S1：Flowchart of participant inclusion.

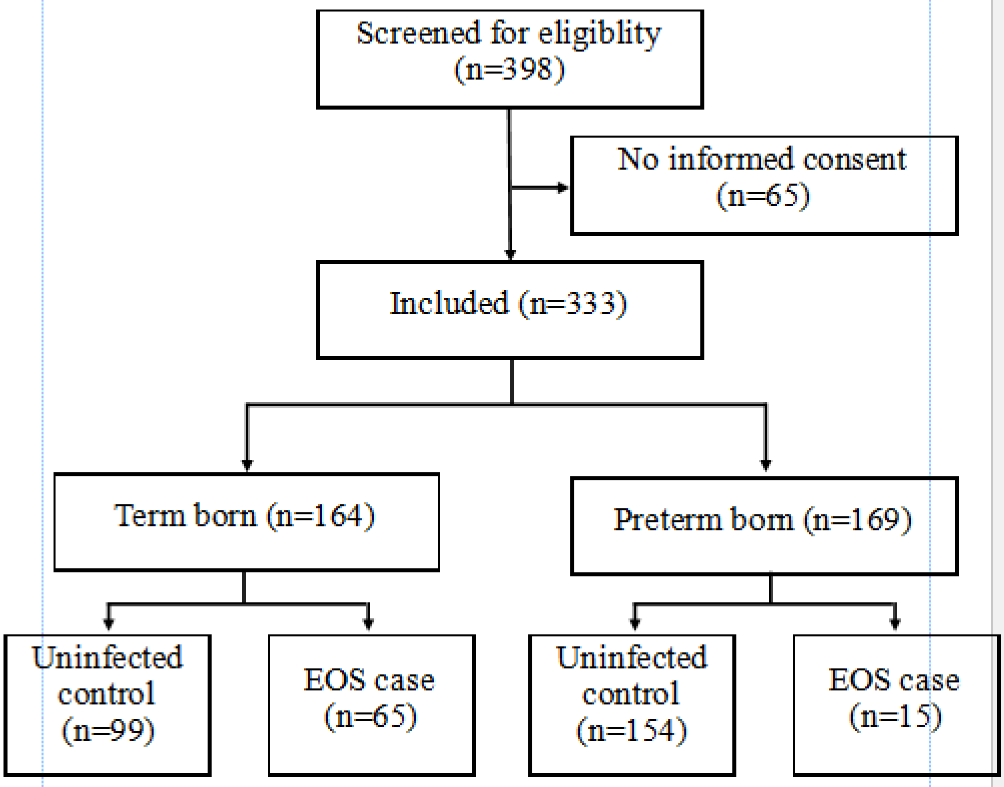

Supplement: Supplementary file 1 [file antibiotics-12-00695-s001.zip › antibiotics-2279337-supplementary.pdf]
